# Molecular regulation of trophoblast stem cell self-renewal and giant cell differentiation by the Hippo components YAP and LATS1

**DOI:** 10.1186/s13287-022-02844-w

**Published:** 2022-05-07

**Authors:** Trishita Basak, Rupasri Ain

**Affiliations:** Division of Cell Biology and Physiology, CSIR-Indian Institute of Chemical Biology, 4, Raja S.C. Mullick Road, Jadavpur, Kolkata, West Bengal 700032 India

**Keywords:** Signaling, Endoreduplication, CDX2, Cell cycle, Kinase, Nuclear size

## Abstract

**Background:**

Trophoblast stem cells (TSCs), the precursors of trophoblast cells of placenta, possess the potential to differentiate into various trophoblastic subtypes in vitro. Establishment of extraembryonic trophoblastic lineage is preceded by the “outside versus inside” positional information in preimplantation embryos, critically synchronized by the Hippo components. Abundant expression of Hippo effector YAP in TSCs and differentiated cells with paucity of information on Hippo regulation of TSC proliferation/differentiation led us test the hypothesis that Hippo dynamics is one of the regulators of  TSC proliferation/differentiation.

**Methods:**

Blastocyst-derived murine TSCs were used. Dynamics of Hippo components were analyzed using immunofluorescence, western blotting, immunoprecipitation, qRT-PCR. Interaction studies were performed using full-length and deletion constructs. BrdU incorporation assay, flow cytometry-based polyploidy analysis and confocal microscopy were used to decipher the underlying mechanism.

**Results:**

YAP translocates to the nucleus in TSCs and utilizes its WW_2_ domain to interact with the PPQY motif of the stemness factor, CDX2. YAP limits TSC proliferation with associated effect on CDX2 target CyclinD1. Trophoblast giant cells (TGC) differentiation is associated with cytoplasmic retention of YAP, heightened pYAP^Ser127^, decrease in the level of the core Hippo component, LATS1, which thereby impedes LATS1-LIMK2 association. Decreased LATS1-LIMK2 complex formation in TGCs was associated with elevated pLIMK2^Thr505^ as well as its target pCOFILIN^Ser3^. Precocious overexpression of LATS1 during trophoblast differentiation decreased TGC marker, *Prl2c2,* diminished pLIMK2^Thr505^ and inactive COFILIN (pCOFILIN^Ser3^) while COFILIN-phosphatase, CHRONOPHIN remained unchanged. LATS1 overexpression inhibited trophoblast endoreduplication with smaller-sized TGC-nuclei, lower ploidy level and disintegrated actin filaments. Inhibition of LIMK2 activity recapitulated the effects of LATS1 overexpression in trophoblast cells.

**Conclusion:**

These results unveil a multilayered regulation of trophoblast self-renewal and differentiation by the Hippo components.

**Supplementary Information:**

The online version contains supplementary material available at 10.1186/s13287-022-02844-w.

## Background

Mechanistic patterning of the trophoblast cells during placentogenesis is reliant upon the self-renewing population of extra-embryonic trophoblast stem cells (TSCs) or the placental progenitor cells that originate from the trophectoderm (TE) layer of the blastocyst. These multipotent cells are committed to differentiate into specialized trophoblastic subtypes of the definitive placenta both in vitro and in vivo in chimeric embryo studies [1]. This lineage-restricted differentiation event predisposes the trophoblast cells to participate in a complex dialogue of diverse functions ensuring lucrative placentation [2]. Mal-differentiation of the “stem” subpopulation of trophoblast precursors leads to chorioallantoic anomalies whose etiologies are underappreciated [3, 4]. Improper development and differentiation of trophoblast cells have been clinically correlated with the pathophysiological conditions arising from placental insufficiencies [5–7] and impose severe consequences on the health of both the mother and the fetus. Indeed, aberrant development of trophoblast cells results in early pregnancy loss and intrauterine lethality or late complications like preeclampsia and intrauterine growth restriction [8, 9]. Therefore, a detailed characterization of trophoblast development would solve the ever-increasing puzzles related to placenta-associated complexities and provide clinical significance to obstetrics.

Deciphering the preliminary events of murine placentation has led to the development of trophoblast stem cell lines which retain the capacity to recapitulate trophoblast-aided placental development ex vivo [10, 11]. These cells can be sustained indefinitely both in a proliferative state as well differentiated to the default giant cell trajectory as a mononuclear polyploid population with nearly 1000 copies of endocycled genomic DNA upon subtle variation in culture conditions [11]. Hippo portrays a conserved kinase cascade [12–14] which operates as an ON–OFF switch to regulate various hallmark processes of stem cell biology like self-renewal [15, 16], proliferation [17], cell cycle regulation [18], migration and differentiation [19, 20]. Although numerous research initiatives have been undertaken to understand Hippo in various tissues and biological processes [21, 22], minimal efforts have been undertaken to appreciate Hippo in reproductive biology. Malfunctioning of Hippo components has been associated with defects in TE as well as inner cell mass (ICM) specification leading preimplantation developmental arrest [23, 24]. Recently, Hippo has been reported to translate positional information into indispensable transcriptional circuits specifying the trophectoderm in preimplantation embryos [25, 26]. Molecular orchestration between Hippo and other signaling pathways is imperative to trophectoderm restriction [27, 28]. Strikingly, differential regulatory inputs converge into equivalent genetic switches to segregate TSCs from its originating trophectoderm layer [29]. Thus, spatiotemporal coordination of diverse regulatory mechanisms structure a particular TE lineage marker at different developmental stages. In line with this, the hierarchical assortment of trophoblast cell fate from a single-layered trophectoderm is interdependent on a complex interplay of genetic cues to equipoise trophoblast self-renewal and differentiation [10, 30], thereby ensuring successful placentation. Expression of YAP in human placenta has been reported by Sun et al., Liu et al. and Saha et al., [31–33]. YAP knockout mice produce lethal phenotypes by E8.5 due to defects in chorioallantoic fusion [34]. Down-regulation of YAP in placentas from preeclamptic mothers has recently been reported [31, 32, 35]. All these lines of evidences conclusively bring out a yet unidentified role of Hippo in trophoblast lineage development, the process critically sustained by a genetic balance between self-renewal and differentiation. Although research initiatives corelating trophoblast dysfunction in placental pathogenesis with the terminal Hippo component YAP has recently begun to be elucidated [36, 37], the dynamicity of the Hippo components and its associated molecular mediators still remains to be explored during development and differentiation of trophoblast cells under normal physiological context. Hence, defining Hippo and the mechanism by which it harmonizes trophoblast self-renewal and differentiation would further sketch the molecular episodes that govern the sublineage pools with temporal replenishment of the trophoblast stem cell hub. Appreciating the cellular mechanisms by which Hippo regulates trophoblast self-renewal and differentiation would provide a comprehensive understanding to fulfill the lacunae in our current knowledge of trophoblast development and provide clinical significance to placental insuffiencies, thereby improving the reproductive success in eutherian mammals by preventing pregnancy related complications.

## Materials and method

### Human sample collection

Human term placental samples were collected from healthy pregnant females and mothers with IUGR babies as described previously [38]. All experimental protocols that used human tissue were approved by Institutional Ethics Committee for Human Research, Calcutta National Medical College, India, in accordance with the relevant guidelines and regulations set forward by Indian Council of Medical Research (http://icmr.nic.in/human_ethics.htm). Informed consent was taken from all human participants in this study.

### Cell culture

Murine blastocyst-derived TSCs (TS_3.5_) were a kind gift from Professor Janet Rossant, The Hospital for Sick Children (SickKids), Toronto, Canada, cultured as previously reported [11, 38–40] and detailed in Additional file 1.

SH-SY5Y cells were obtained from American Type Culture Collection (USA) and grown as per ATCC instructions. All details pertaining to SH-SY5Y culture are documented in Additional file 1.

### Cloning and characterization of full-length mouse YAP, CDX2, LATS1 and deletion constructs of YAP and CDX2

Cloning of full-length mouse YAP (NM_001171147.1), CDX2 (NM_007673.3) and LATS1 (NM_010690.1) has been described in detail in Additional file 1. Cloning and characterization of the deletion constructs of YAP (ΔWW_1,_ ΔWW_2_ and ΔWW_1_WW_2_) and CDX2 (ΔCDX2) is detailed in Additional file 1. All the primers and restriction enzymes used for cloning are listed in Table 1.

**Table 1:** List of primers and restriction enzymes used for cloning of various full-length and deleted constructs

Clone identity	Fragment	Primer sequence (5′–3′)	Restriction enzyme
*Fl-Yap*	–	AATAGGTACCAATGGAGCCCGCGCAACAG	KpnI
		AATAGGATCCCTATAACCACGTGAGAAAG	BamHI
ΔW_1_	F1	AATAGGTACCAATGGAGCCCGCGCAACAG	KpnI
		AATAGCTAGCATCATCAGGGATCTCAAA	NheI
	F2	AATAGCTAGCGCCATGCTTTCGCAACTG	NheI
		AATAGGATCCCTATAACCACGTGAGAAAG	BamHI
ΔW_2_	F1	AATAGGTACCAATGGAGCCCGCGCAACAG	KpnI
		AATTGCATGCTGAGGCAGAATTCATCAGCG	PaeI
	F2	AATAGCATGCCTGGACCCAAGGCTGGAC	PaeI
		AATAGGATCCCTATAACCACGTGAGAAAG	BamHI
ΔW_1_W_2_	F1	AATAGGTACCAATGGAGCCCGCGCAACAG	KpnI
		AATAGCTAGCATCATCAGGGATCTCAAA	NheI
	F2	AATAGCTAGCGCCATGCTTTCGCAACTG	NheI
		AATTGCATGCTGAGGCAGAATTCATCAGCG	PaeI
	F3	AATAGCATGCCTGGACCCAAGGCTGGAC	PaeI
		AATAGGATCCCTATAACCACGTGAGAA AG	BamHI
*Fl-Cdx2*	–	AATAAAGCTTATGTACGTGAGCTACCT	HindIII
		AATTGGTACCTCACTGGGTGACAGTGG	KpnI
*ΔCdx2*	F1	AATAAAGCTTATGTACGTGAGCTACCT	HindIII
		AATACTCGAGGTTCAGGCCGCCGGAGTG	XhoI
	F2	AATACTCGAGCAGTCCCCAGGGCCATCC	XhoI
		AATTGGTACCTCACTGGGTGACAGTGG	KpnI
*Fl-Lats1*	–	AATAGCGGCCGCAATGAAGAGGGGTGAAAAG	NotI
		ATATGGTACCTAAACATACACTAGATCTCGGT	KpnI

### Transient transfection of siRNAs and plasmid

Down-regulation of endogenous YAP was performed by transfection of two pre-validated silencer select siRNAs [(s202423 and s76160), Ambion] using Lipofectamine RNAiMax (Invitrogen) as per standard protocol which has been described in detail in Additional file 1.

For overexpression and interaction studies, full-length or deleted constructs were transfected using Lipofectamine 2000 (Invitrogen) as per manufacturer’s protocol. Details pertaining to ectopic expression-based functional and interaction studies are described in detail in Additional file 1

### BMS-3 treatment

For inhibition of endogenous LIMK2 in differentiated trophoblast cells, the pharmacological inhibitor BMS-3 (HY-18304, Med Chem Express) was used. Trophoblast cells were seeded under differentiating conditions and allowed to adhere. Cells were then treated with three different doses of BMS-3 (2.5 µM, 5 µM, 10 µM) for 6 h. Control cells were treated with equivalent amount of DMSO. Cells were allowed to differentiate post-treatment and harvested 72 h thereafter.

### RNA extraction, reverse transcription, PCR and quantitative real-time PCR

Isolation of total RNA, reverse transcription and qPCR was done as per standard protocol and is detailed in Additional file 1. Primers used for qPCR studies are listed in Table 2.

**Table 2:** List of primers used for quantitative real-time PCR

Gene name	Accession no		Primer sequence (5′–3′)
*Yap*	NM_001171147.1	Fwd	GGAGAGACTGCGGTTGAAACA
		Rev	TTCGGAGTCCCTCCATCCTG
*Cyclin D1*	NM_007631	Fwd	GTGCGTGCAGAAGGAGATTGTG
		Rev	GGGCTCCAGGGACAGGAA
*Lats1*	NM_010690.1	Fwd	CTGAAGTGCTACTGCGAACA
		Rev	GAGAAGTTTGCCAGTTGATAACC
*Prl2c2*	NM_031191	Fwd	CATCTCCAAAGCCACAGACATAA
		Rev	TGAATGCGAGCAGCTTCATTG
*rPL7*	NM_011291	Fwd	AAGAAGCGGATTGCCTTGAC
		Rev	TAACTTGAAGGGCCACAGGAA

### Western blot analysis

Western blotting was performed as described previously [38–42]. The procedure and image acquisition have been described in detail in Additional file 1.

### Immunoprecipitation

For immunoprecipitation, 250 μg cell lysate was incubated overnight at 4ºC with the desired capture antibody and immunoprecipitation was performed as described previously [38]. Details pertaining to immunoprecipitation are described in Additional file 1.

### Nuclear and cytoplasmic fractionation

Lysates from TSCs and day 6-differentiated trophoblast cells were fractionated using Cell Fractionation kit (Cell Signaling Technologies) as per manufacturer’s protocol. Fractions were quantified for their total protein content and 5 μg of individual fractions were resolved under denaturing conditions and probed with YAP antibody. GAPDH and Histone H3 were used as the cytoplasmic and nuclear marker, respectively, at recommended dilution.

### Immunofluorescence

Immunofluorescence was performed as described previously [40] and is detailed in Additional file 1.

### Antibodies

A list of antibodies used in this study is given in Table 3.

**Table 3:** List of antibodies used for experimental purposes

	Antibody	Company (Catalog no.)	Dilution
1	Mouse monoclonal anti-YAP	Cell Signaling Technology (12,395)	1:1000 (WB),,1:200 (IP), 1:400 (IF)
2	Rabbit monoclonal anti-phospho YAP^ser127^	Cell Signaling Technology (130,084)	1:1000 (WB)
3	Rabbit polyclonal anti-CDX2	Cell Signaling Technology (3977)	1:1000 (WB)
4	Rabbit monoclonal anti-LATS1	Cell Signaling Technology (3477)	1:1000 (WB), 1:100 (IP)
5	Rabbit polyclonal anti-phospho LATS1^Thr1079^	Cell Signaling Technology (8654)	1:1000 (WB)
6	Rabbit monoclonal anti-COFILIN	Cell Signaling Technology (5175)	1:1000 (WB)
	Rabbit monoclonal anti-phospho COFILIN^Ser3^	Cell Signaling Technology (3313)	1:1000 (WB)
7	Rabbit polyclonal anti-CYCLIN D1	Cell Signaling Technology (2922)	1:250 (WB)
8	Rabbit polyclonal anti-MST1	Cell Signaling Technology (3682)	1:1000 (WB)
9	Rabbit monoclonal anti-phospho MST1^Thr183^	Cell Signaling Technology (49,332)	1:1000 (WB)
10	Rabbit monoclonal anti-CHRONOPHIN	Cell Signaling Technology (4686)	1:1000 (WB)
11	Rabbit monoclonal anti-GAPDH antibody	Cell Signaling Technology (5174)	1:2000 (WB)
12	Rabbit monoclonal anti-Histone H3	Cell Signaling Technology (34,499)	1:2000 (WB)
13	Rabbit polyclonal anti-LIMK2	Sigma-Aldrich (HPA008183)	1:250 (WB), 1:50 (IP)
14	Rabbit polyclonal anti-LIMK2^Thr505^	Cell Signaling Technology (3841)	1:1000 (WB)
15	Mouse monoclonal anti-FLAG	Sigma-Aldrich (F3165)	1: 1000 (IP)
16	HRP-conjugated anti-rabbit IgG	Cell Signaling Technology (34,499)	1:2000 (WB)
17	HRP-conjugated anti-rabbit IgG	Cell Signaling Technology (34,499)	1:2000 (WB)
18	TRITC conjugated goat anti-mouse IgG	Sigma-Aldrich (F3165)	1:2000 (IF)

### Hoechst and Phalloidin staining of F-actin

Hoechst and Phalloidin staining were performed as described previously [40]. Experimental details pertaining to Hoechst and Phalloidin staining under LATS1 overexpressing and LIMK2 inhibited conditions are described in Additional file 1.

### 5-Bromodeoxyuridine incorporation assay

BrdU incorporation assay was performed as described previously [43] and is detailed in Additional file 1.

### Polyploidy analysis by flow cytometry

Flow cytometry-based analysis of polyploidy was performed as described previously [40] and is decribed in Additional file 1.

### Statistical analysis

Comparison of independent means for all the data presented was made using Student's unpaired *t*-test in at least three independent biological replicates using the software GraphPrism7. For all experiments, *p* value of 0.05 was considered as the highest borderline of statistical significance and *p* < 0.05 was considered as significant and is marked with asterisk(s) in the study.

## Results

### Nucleo-cytoplasmic shuttling of the terminal Hippo component YAP signifies its function in trophoblast differentiation

**Fig. 1:**
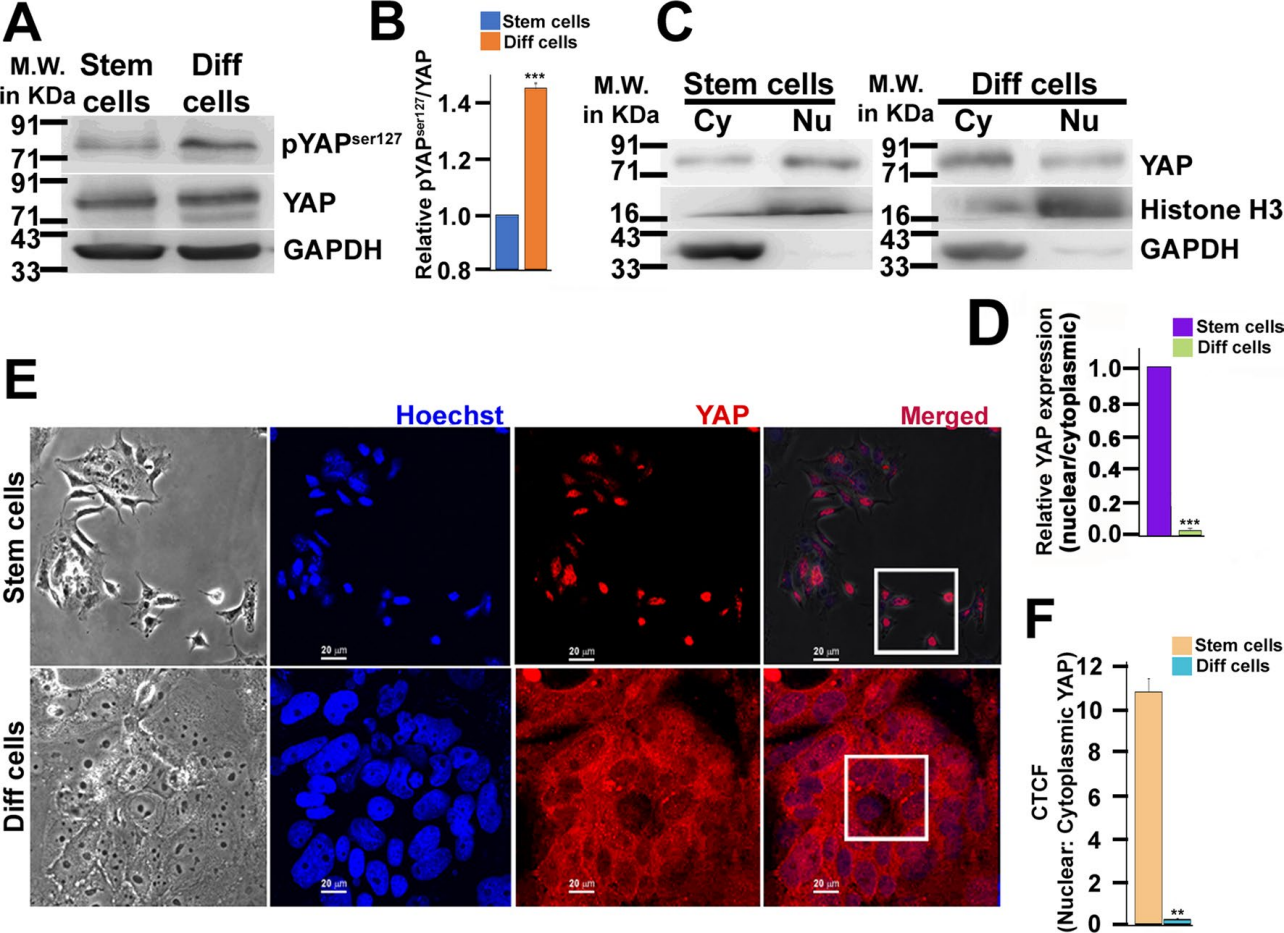
YAP translocate to the nucleus in trophoblast stem cells. **A** Western blot analysis of YAP and pYAP^ser127^ using cell lysates prepared from TSCs (stem cells) and differentiated cells. GAPDH was used as an endogenous control. **B** Densitometric analysis of protein bands from **A** using NIH ImageJ software. **C** Western blot analysis of YAP in cytoplasmic and nuclear fraction of TSCs (stem cells) and differentiated cell lysate. GAPDH and Histone H3 were used as the cytoplasmic and membrane marker, respectively. **D** Densitometric quantification of YAP bands from **C** showing the relative amount of YAP in the nucleus and cytoplasm of TSCs and differentiated trophoblast cells. Normalization of nuclear and cytoplasmic YAP was done using Histone H3 and GAPDH, respectively. **E** Confocal photomicrographs of YAP (red) immunostained in TSCs and trophoblast cells on the 6th day of differentiation. Boxed areas on the right panel show extensive localization of YAP in the nucleus of TSCs and cytoplasm of differentiated cells. The nuclei were counterstained using Hoechst (blue). Scale bar: 20 µm. Magnification: × 60. **F** Ratio of the mean fluorescence intensity in the nucleus relative to the cytoplasm of TSCs and differentiated cells from **E** is represented as Corrected Total Cell Fluorescence (CTCF). Values are represented as mean ± SEM from three independent biological replicates and *n* = 10 in each replicate. **p < 0.005; ***p < 0.0005

To understand the effect of Hippo dynamics on trophoblast differentiation, the phosphorylation status of YAP at serine 127 residue, which is crucial for its localization and activity [44], was initially analyzed using cell lysates from TSCs and differentiated trophoblast cells by western blotting (Fig. 1A). The extent of ser127 phosphorylation, which promotes cytoplasmic sequestration was enhanced significantly (*p* < 0.0005) by 45% when TSCs were induced to differentiate by withdrawal of mitogens (Fig. 1B). Low levels of pYAP^ser127^ in TSCs indicated a plausible translocation of YAP into the nucleus. This predicted nuclear shuttling of YAP in TSCs and its cytoplasmic retention upon differentiation was affirmed by fractionating cell lysates from TSC and differentiated trophoblast cells into nuclear and cytoplasmic fractions followed by western blotting. In line with our prediction, YAP was found to be enriched in the nuclear fraction of TSCs, whereas enrichment of YAP was observed in the cytoplasmic fraction of differentiated trophoblast cells (Fig. 1C), with densitometric analysis showing significant (*p* < 0.0005) YAP enrichment in the nucleus of TSCs as compared to differentiated cell (Fig. 1D). To confirm nucleo-cytoplasmic shuttling of YAP in TSCs and differentiated cells using another line of evidence, cellular localization of YAP in TSCs and differentiated trophoblast cells was assessed by immunofluorescence staining. Localization of YAP in nucleus of TSCs and cytoplasmic retention of YAP in differentiated trophoblast cells was evident from immunostaining (Fig. 1E). The relative nuclear: cytoplasmic intensity of YAP was reduced by almost 10-folds (*p* < 0.005) in differentiated trophoblast cells (Fig. 1F). Therefore, these data confirm that YAP accumulates in the nucleus of TSCs and is primarily retained in the cytoplasm in differentiated trophoblast cells. Thus, nuclear YAP might have some yet unknown function in TSCs.

### Function of YAP in TSC self-renewal

**Fig. 2:**
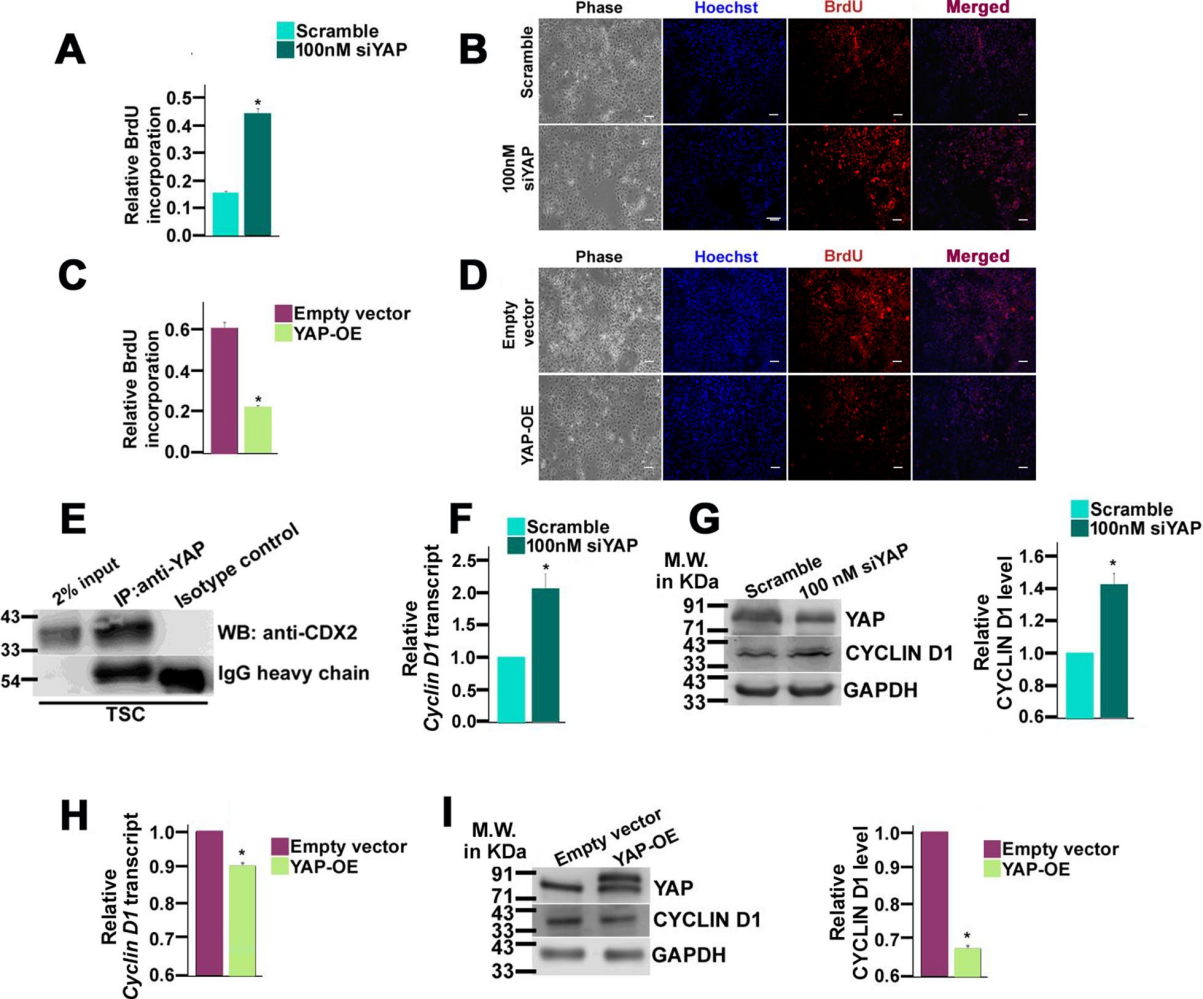
YAP restricts trophoblast stem cell proliferation. **A** BrdU incorporation assay in TSCs transfected with scramble or YAP siRNA. **B** Photomicrographic images of BrdU incorporated cells (red) following transfection with either scramble siRNA or YAP siRNA. Cells stained with Hoechst (blue) to mark all nuclei. Scale bar: 25 µm. Quantitation of the fluorescence intensity of BrdU has been represented on the right. **C** BrdU incorporation assay in TSCs following transfection with either empty vector backbone or YAP cDNA. **D** Photomicrographic images of BrdU incorporated cells (red) following transfection with either empty vector backbone or YAP cDNA. Cells were stained with Hoechst (blue) to mark all nuclei. Scale bar: 25 µm. Fluorescence intensity of BrdU has been quantified and represented on the right. Normalization was done against the cell number (CTCF/Cell) in three different biological replicates. **E** Coimmunoprecipitation of endogenous CDX2 with YAP using TSC lysate. Immunoprecipitation (IP) with anti-YAP antibody was followed by western blot with anti-CDX2 antibody. IP using an isotype matched antibody was used as a negative control. IgG heavy chain detected in each sample was used as loading control. **F** Quantitative real-time PCR analysis of *Cyclin D1* transcripts in TSCs transfected with either scramble or YAP siRNA. **G** Western blot analysis of CYCLIN D1 using cell lysates from TSCs transfected with either scramble or YAP siRNA. GAPDH was used as an endogenous control. Densitometric analysis of the protein bands from **G** using NIH ImageJ is shown with the bar graphs on the right side of the blots. **H** Quantitative real-time PCR analysis of *Cyclin D1* transcripts in TSCs transfected with vector back bone or YAP cDNA. **I** Western blot analysis of CYCLIN D1 using cell lysates from TSCs transfected with vector back bone or YAP cDNA. GAPDH was used as an endogenous control. Densitometric analysis of the protein bands from **I** using NIH ImageJ is shown with the bar graphs on the right side of the blots. Values are represented as mean ± SEM from three independent biological replicates. Statistical analysis was performed using Student’s unpaired *t*-test. *p < 0.05; **p < 0.005

To analyze the effect of nuclear YAP on the proliferative potential of TSCs, loss of function of YAP using RNA interference and gain in function using ectopic overexpression of YAP cDNA were used in BrdU incorporation assay. Endogenous YAP was transiently down-regulated using two pre-validated Silencer Select siRNA which targets the coding sequence of YAP at two different exons. An optimum concentration of 100 nM siRNA cocktail (50 nM each siRNA) was selected depending on the maximum down-regulation of *Yap* transcript observed in a dose response experiment (Additional file 2: Fig. S1a). At a seeding density of 50,000 cells, knockdown of endogenous YAP significantly enhanced proliferation and the incorporation of BrdU by 40% (*p* < 0.05) onto the newly synthesized DNA strands (Fig. 2A). BrdU incorporation followed by immunofluorescence staining showed enhanced BrdU immunofluorescence upon RNA interference of YAP as compared to scramble siRNA-treated TSCs (Fig. 2B). To reaffirm YAP-dependent proliferation of TSCs, YAP was overexpressed in TSCs and the extent of overexpression was confirmed by qPCR (Additional file 2: Fig. S1b). As anticipated, an elevation in the level of YAP significantly inhibited BrdU incorporation (Fig. 2C) resulting in lesser BrdU immunofluorescence as compared to empty vector transfected control (Fig. 2D). For both RNA interference and cDNA overexpression, YAP restricted trophoblast proliferation at a density of 50,000 cells. However, when cells were seeded at a lower density (10,000 cells), similar results were not obtained (data not shown). Thus, it appears that YAP at higher cell density, a scenario that mimics *in vivo* conditions, where cells are in physical contact with each other, restricts trophoblast proliferation.

YAP being a transcription cofactor lacks the necessary DNA-binding domain. Therefore, it must associate with transcription factors to reinforce its function. In search of trophoblast specific transcription factors capable of interacting with YAP, an in-depth analysis of mouse CDX2 and YAP protein sequences was performed. Proteins containing a WW domain can physically interact with PPxY (x = any amino acid) motifs present in other proteins [45]. Interestingly, the amino acid sequence of CDX2 harbors a PPQY motif between amino acid residues 34–37 which can serve as a putative-binding site for the two WW domains present in YAP. YAP was found to form an immune complex with CDX2 indicating their physical interaction (Fig. 2E). CDX2 is known to transactivate CYCLIN D1 in TSCs [43]. Therefore, to understand YAP-reliant self-renewal of TSCs, *Cyclin D1* transcript and protein levels were assessed in TSCs in YAP knocked down and overexpressed conditions. Knock down of endogenous YAP and maintenance of stemness for 48 h led to a significant increase (*p* < 0.05) in *Cyclin D1* transcript (Fig. 2F) and protein level (Fig. 2G). Ectopic overexpression of YAP significantly reduced *Cyclin D1* transcript (Fig. 2H) and protein levels (Fig. 2I). To corelate the above findings with placentation defects in humans, the level of YAP was analyzed in the development of IUGR, a pathophysiological condition characterized by reduced trophoblast proliferation [46]. In agreement with the findings of this study, the level of YAP was higher in IUGR placentas as compared to normal placentas (Additional file 2: Fig. S3). Collectively, these data demonstrate that nuclear YAP restricts murine TSC proliferation by potentially sequestering CDX2 and inhibiting CYCLIN D1.

### WW_2_ domain of YAP is involved in interaction with the PPQY motif of CDX2.

**Fig. 3:**
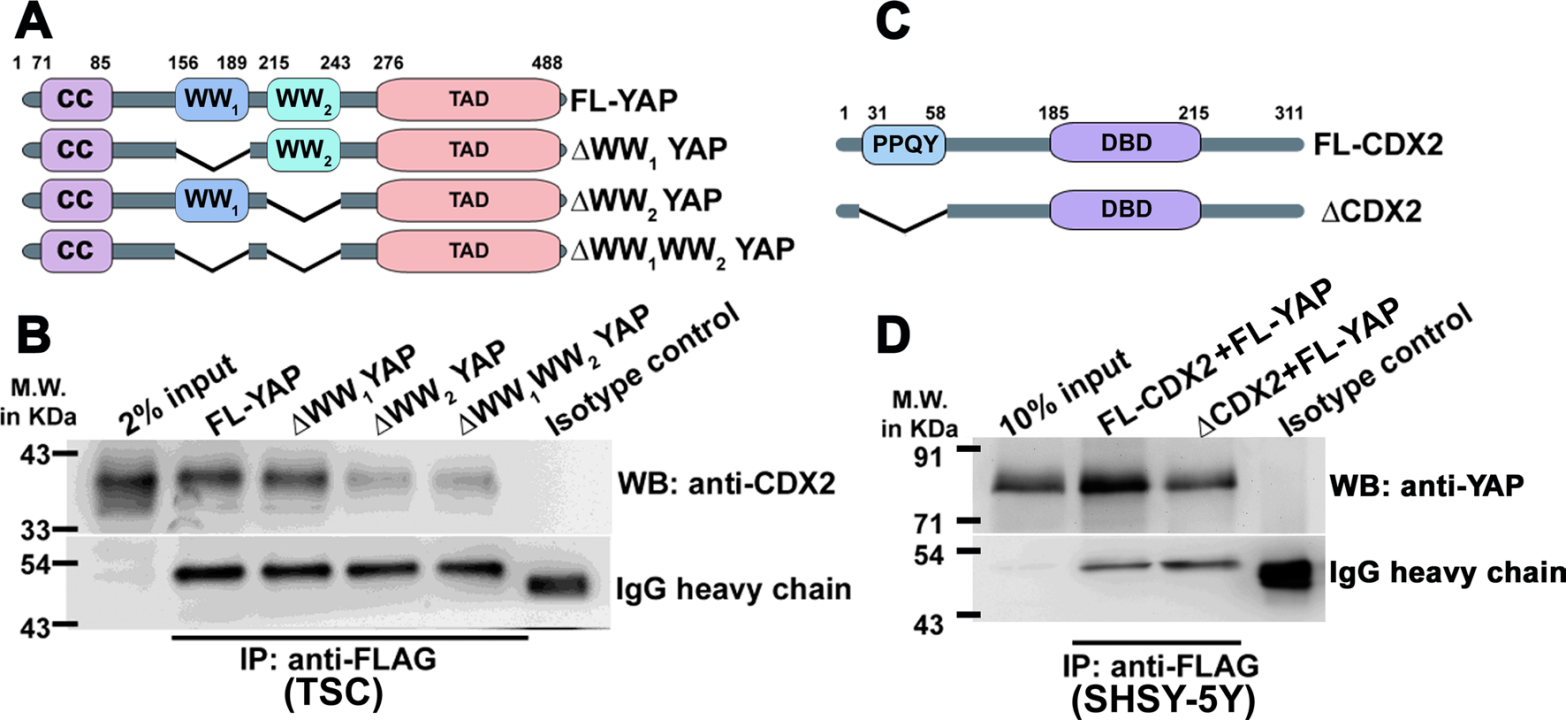
WW_2_ domain of YAP and PPQY motif of CDX2 are involved in YAP-CDX2 interaction. **A** Schematic illustration of the domain organization of YAP showing the various deletion constructs cloned and used for experimental purposes. The amino acid residue numbers are indicated at the top of each construct. CC, Coil–coil domain; TAD, transactivation domain; WW, tryptophan-rich domains. **B** Immunoprecipitation of CDX2 with either FLAG-tagged full-length YAP or various deletion constructs of YAP using lysates from TSCs transfected with either FLAG-tagged full-length YAP or various deletion constructs of YAP. Immunoprecipitation was done using anti-FLAG antibody followed by immunoblot with anti-CDX2 antibody. Immunoprecipitation using an isotype matched antibody was used as a negative control. IgG heavy chain was used as loading control. **C** Schematic illustration of the domain organization of CDX2 showing the region deleted for the experimental purpose. The amino acid residue numbers are indicated at the top of each construct. DBD, DNA-binding domain. **D** Either full-length CDX2 or ΔCDX2 was transfected in SH-SY5Y cells along with full-length YAP and maintained for 48 h. Extracts were immunoprecipitated using anti-FLAG antibody and coimmunoprecipitation of YAP was analyzed using anti-YAP antibody. Total cell lysates were used as input samples in **B** and **D**

In order to scrutinize the functional domain utilized in YAP-CDX2 interaction, three domain deletion mutants of YAP were constructed which encompassed a deletion of WW_1_ (Δ156-189 amino acids), WW_2_ (Δ215-243 amino acids) and WW_1_WW_2_ (Δ156-189 amino acids and Δ215-243 amino acids), respectively (Fig. 3A). Following cloning and sequencing, the expression of the mutants was tested by transfection in SH-SY5Y cells. Due to small change in the molecular weight of these mutants, a shift in protein band shift could not be detected by immunoblot analysis (Additional file 2: Figure S2a). Expression of deletion mutants were therefore confirmed by a band shift in PCR (Additional file 2: Figure S2b) following reverse transcription. To test which domain of YAP is involved in interaction with CDX2, either full-length YAP or individual YAP deletion constructs were ectopically expressed in TSCs. Interestingly, both full-length YAP and ΔWW_1_YAP coimmunoprecipitated with CDX2 in TSCs. On the contrary, coimmunoprecipitation of ΔWW_2_YAP and CDX2 was severely compromised. Deletion of both WW_1_ and WW_2_ yielded similar results (Fig. 3B). Thus, a functional YAP-CDX2 interaction requires WW_2_ domain of YAP in TSCs.

To specify the region of CDX2 utilized in YAP-CDX2 interaction, PPQY deletion mutant of CDX2 (Δ31-58) was constructed (Fig. 3C) and confirmed by a band shift in RT-PCR and immunoblot in SH-SY5Y cells (Additional file 2: Fig. S2c and d). However, owing to the abundance of endogenous CDX2 in TSCs, we expressed the constructs along with FL-YAP in SH-SY5Y cells which lacked both endogenous YAP and CDX2. PPQY deletion led to decreased YAP-CDX2 interaction in SH-SY5Y cells (Fig. 3D). Results from these experiments show association of YAP and CDX2. Furthermore, YAP-CDX2 binding involves interaction between the WW_2_ domain of YAP and PPQY motif of CDX2.

### Hippo component LATS1 is involved in regulation of trophoblast giant cell differentiation.

**Fig. 4:**
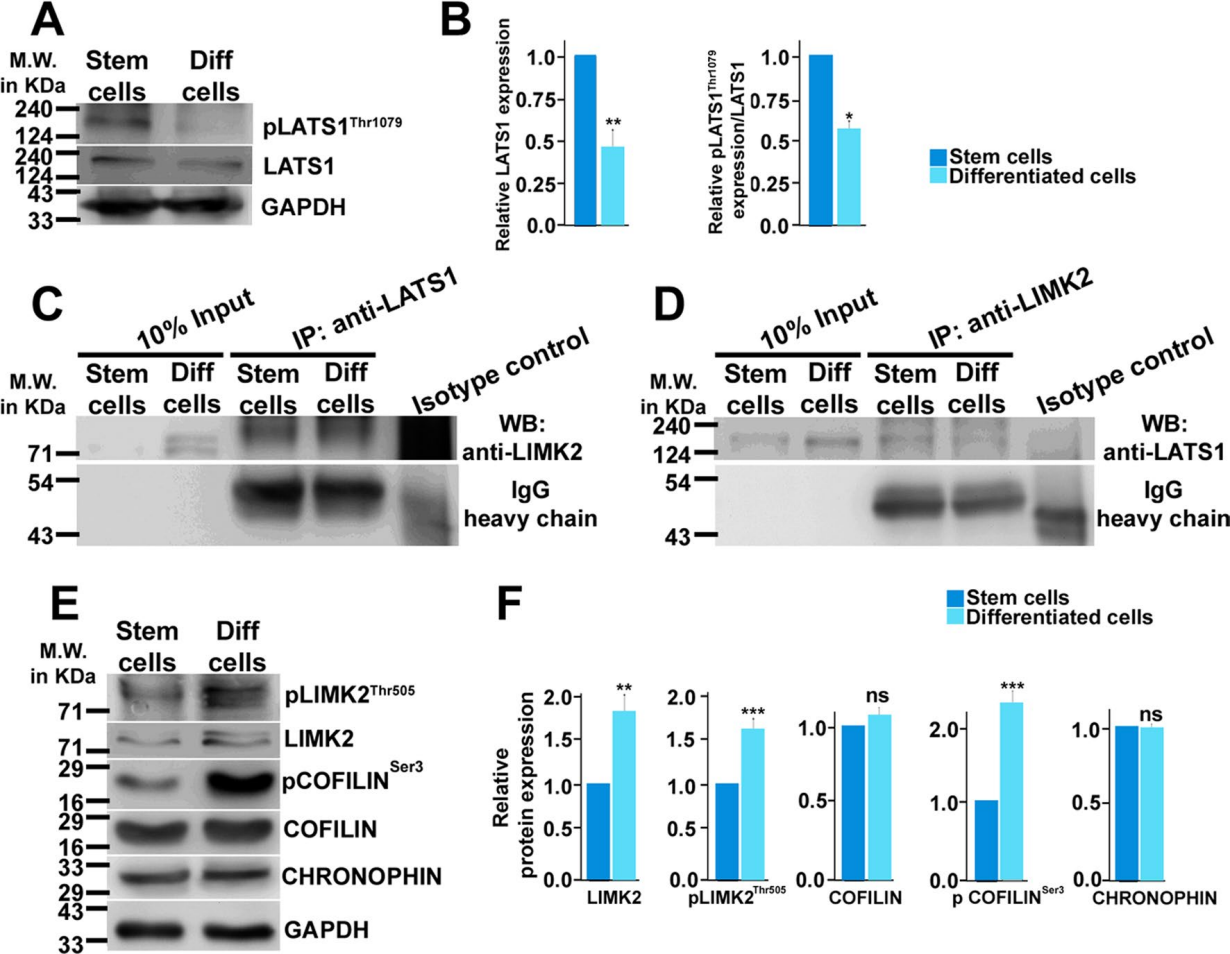
Role of LATS1 in trophoblast giant cell differentiation. **A** Western blot analysis of LATS1 and pLATS1^Thr1079^ in TSCs (Stem cells) and differentiated trophoblast cells. GAPDH was used as an internal control. **B** Densitometric quantification of the protein bands from **A**. The level of pLATS1^Thr1079^ was quantified relative to the basal level of LATS1. **C** Western blotting showing the coimmunoprecipitation of endogenous LATS1 with LIMK2 in TSCs and differentiated trophoblast cells. Immunoprecipitation (IP) with anti-LATS1 antibody was followed by immunoblot with anti-LIMK2 antibody. Immunoprecipitation using an isotype matched IgG was used to ensure the specificity of the capturing antibody. **D** Immunoprecipitation demonstrates that LATS1 exists as a protein complex pulled down by anti-LIMK2 antibody. **E** Western blot analysis showing the probing of LIMK2, pLIMK2^Thr505^, COFILIN, pCOFILIN^Ser3^ and CHRONOPHIN in TSCs and differentiated trophoblast cells. **D** Densitometric analysis of the protein bands in **E** using GAPDH as an endogenous control. LIMK2, COFILIN and CHRONOPHIN are normalized relative to the level of the housekeeping gene, whereas the level of pLIMK2^Thr505^ and pCOFILIN^Ser3^ was normalized relative to the basal level of LIMK2 and COFILIN, respectively. Values are represented as mean ± SEM from three independent biological replicates. Statistical analysis was performed using Student’s unpaired *t*-test, *p < 0.05; **p < 0.005; ***p < 0.0005, ns, nonsignificant

To further illustrate the influence of the core Hippo components on trophoblast differentiation, the relative abundance of LATS1 in TSCs and differentiated trophoblast cells was analyzed. Induction of trophoblast differentiation was associated with a significant reduction in LATS1 as well as pLATS1^Thr1079^ levels (Fig. 4A, B). Interestingly, the expression dynamics of LATS1 parallels to the phosphorylation status of upstream Hippo kinase MST1 at Thr183 (signal for autophosphorylation and activation) which is known to phosphorylate LATS1 at Thr1079 (Additional file 2: Fig. S4). However, a decrease in the pool of total LATS1 prompted us to understand the relevance of decreased LATS1 in trophoblast differentiation. A role of LATS1/2 in regulating cytokinetic cellular changes has been reported [47]. In addition, LATS1 has been reported to bind to LIM domain containing proteins [48, 49]. Screening of expression of LIMK1 and LIMK2 in TSCs and differentiated trophoblast cells showed that not LIMK1 but LIMK2 is expressed in trophoblast cells (data not shown). LATS1 was found to coimmunoprecipitate with LIMK2 in trophoblast cells (Fig. 4C) and was reaffirmed using reverse coimmunoprecipitation of LIMK2 followed by western blotting with LATS1 (Fig. 4D). LATS1 is known to inhibit phosphorylation of COFILIN by LIMK1 [49]. LIMK2 deletion was shown to cause compromised COFILIN phosphorylation along with a reduced F/G-actin ratio in airway smooth muscle cells [50]. In addition, CHRONOPHIN (phosphatase) is known to regulate the levels of COFILIN phosphorylation status [51]. Our data on association of LATS1 and LIMK2, which is more prominent in TSCs as compared to differentiated trophoblast cells, led us to further investigate the abundance and dynamicity of LIMK2, its phosphorylated form, pLIMK2^Thr505^, COFILIN, phospho-COFILIN and CHRONOPHIN in TSCs and differentiated trophoblast cells (Fig. 4E). Interestingly, the level of LIMK2 protein was significantly elevated upon trophoblast differentiation. Also, a significant pool of LIMK2 was found to be phosphorylated as pLIMK2^Thr505^ in differentiated cells (Fig. 4E, F). Heightened LIMK2 was associated with a robust increase of pCOFILIN^Ser3^ which may be corelated with increased LIMK2 activity upon trophoblast differentiation (Fig. 4E, F). On the contrary, the level of unphosphorylated COFILIN pool remained unaltered upon trophoblast differentiation. Further, the level of CHRONOPHIN was unaffected by differentiation of trophoblast cells (Fig. 4E, F). Altogether, our data demonstrate a potent LATS1-LIMK2 association in TSCs. Reduced LATS1 in differentiated cells might allow active LIMK2 (pLIMK2^Thr505^) to phosphorylate its substrate (pCOFILIN^Ser3^).

### Endoreduplication of trophoblast giant cells is potentiated by decreased LATS1

**Fig. 5:**
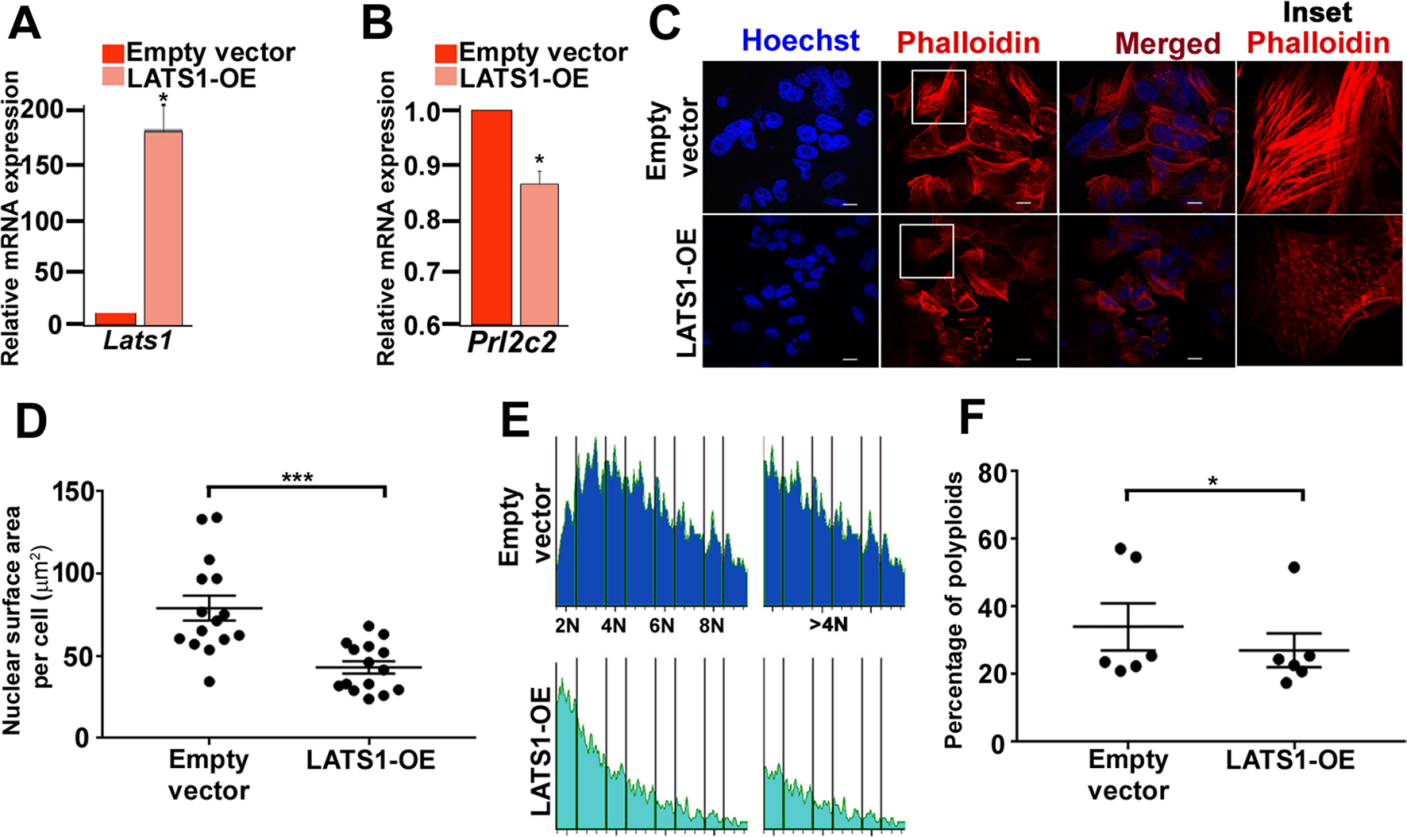
Limited LATS1 potentiates trophoblast endoreduplication. **A** Quantitative real-time PCR analysis of LATS1 overexpression in trophoblast cells. Differentiation was induced 6 h post-transfection and the cells were harvested after 72 h. **B** Quantitative real-time PCR analysis of *Prl2c2* in LATS1 overexpressing cells under similar conditions described in **A**. **C** Confocal photomicrographic images of LATS1 overexpressing cells 72 h post-differentiation induction. The nuclei have been stained using Hoechst (blue) and counterstaining of the cytosolic actin filaments has been done using DyLight™ 554 Phalloidin (red). Boxed areas represent disintegration of the actin filaments which has been magnified in the rightmost panel. Scale bar: 10 µm. Magnification: × 88 **D** Quantitative assessment of the nuclear surface area/cell of trophoblast cells upon LATS1 overexpression using ImageJ software. **E** FACS profile showing the DNA content histogram (top) of the gated population in cells transiently transfected with LATS1 or empty vector control and stained with Hoechst. The percentage of cells with DNA content > 4 N is shown in the right panel. **E** The percentage of polyploid nuclei has been plotted from an average of six independent biological flow cytometric replicates. Values are represented as mean ± SEM from three independent biological replicates. Statistical analysis was performed using Student’s unpaired *t*-test, *p < 0.05; **p < 0.005; ***p < 0.0005

To understand the functional significance of change in LATS1 expression upon TSC differentiation, LATS1 was overexpressed in TSCs followed by induction of differentiation and cells were harvested on day 3. The extent of overexpression of *Lats1* transcript in comparison with empty vector transfected cells was confirmed by quantitative real-time PCR (Fig. 5A). Expression of trophoblast giant cell (TGC) marker, *Prl2c2,* was reduced by 15% in LATS1 overexpressing cells as compared to vector control (Fig. 5B) indicating compromised TGC formation in the presence of excess LATS1. To morphologically ascertain LATS1-mediated phenotypic changes in TGCs, *Lats1* overexpression was followed by Hoechst–Phalloidin staining (Fig. 5C). Interestingly, LATS1 overexpression led to reduction in the size of the nuclei as compared to the vector control (Fig. 5C, D). The F-actin filaments were observed by staining the cells with DyLight™ 554 Phalloidin. In cells treated with empty vector backbone, the F-actin filaments exhibited a regular arrangement which were evenly distributed throughout the cytoplasm. On the contrary, F-actin filaments appeared disintegrated in LATS1 overexpressing trophoblast cells (Fig. 5C, last panel columns).

LATS1-mediated impediment of TGC formation was further confirmed by quantitative assessment of polyploid cells by FACS analysis. Differentiating trophoblast cells on day 3 in the absence or presence of ectopically overexpressed LATS1 were used for this assay. Cells that have undergone endoreduplication were detected as peaks > 4n and ≤ 10n in the FACS profiles. Ectopic LATS1 expression limited the number of cells with > 4 N DNA content (Fig. 5E). There was an overall 8% reduction in the percentage of polyploid cells in LATS1 overexpressing cells as compared to control cells (Fig. 5F). All these lines of evidences conclusively suggest that LATS1 suppresses the formation of endoreduplicated TGCs.

### Ectopic overexpression of LATS1 is associated with decreased COFFILIN activation

**Fig. 6:**
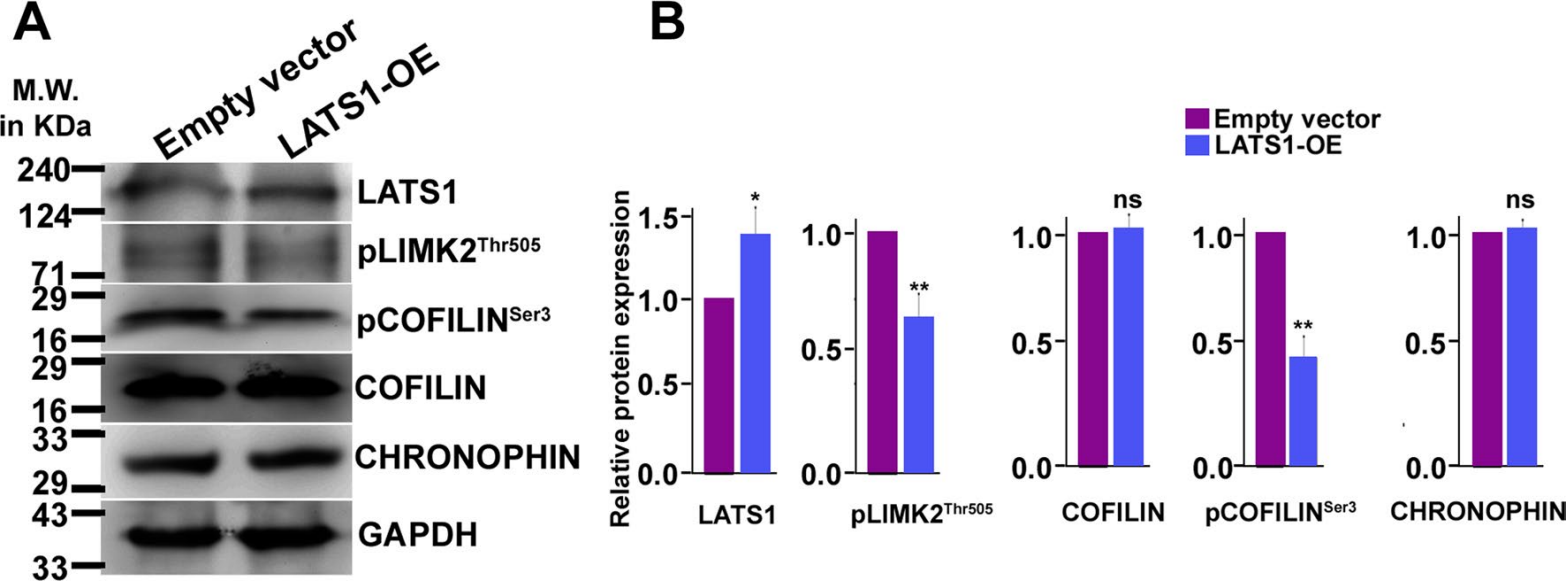
LATS1 influences COFILIN phosphorylation. **A** Western blot analysis of pLIMK2^Thr505^, COFILIN, pCOFILIN^Ser3^ and CHRONOPHIN in trophoblast cells ectopically overexpressing LATS1 as compared to empty vector transfected cells. **B** Densitometric analysis of the protein bands from A. Band intensities of COFILIN and CHRONOPHIN were normalized using GAPDH as the loading control, whereas the level of pLIMK2^Thr505^ and pCOFILIN^Ser3^ was normalized relative to the basal level of the protein. Error bars shows the SEM of biological triplicate runs. Statistical analysis was performed using Student’s unpaired *t*-test, *p < 0.05; **p < 0.005, ns, nonsignificant

Interaction of LATS1 with LIMK2 and associated COFILIN phosphorylation/inactivation upon trophoblast differentiation prompted us to explore cofilin dynamics upon manipulation of LATS1. LIMK2 activation and COFILIN inactivation as marked by pLIMK2^Thr505^ and pCOFILIN^Ser3^ was significantly decreased upon LATS1 overexpression as compared to empty vector transfected cells (Fig. 6A, B). However, the basal level of COFILIN remained unchanged. Additionally, no change in the expression of CHRONOPHIN, a phosphatase responsible for dephosphorylating COFILIN was observed (Fig. 6A, B). Reduced phosphorylation of COFILIN in LATS1 overexpressing cells can thus be attributed to limited LIMK2 activity by plausible sequestration by LATS1 and not a primary effect of CHRONOPHIN-mediated COFILIN dephosphorylation. Taken together, these data suggest that precocious expression of LATS1 limit LIMK2 activation and subsequent COFILIN phosphorylation.

### Pharmacological inhibition of LIMK2 recapitulates the effect of LATS1 overexpression

**Fig. 7:**
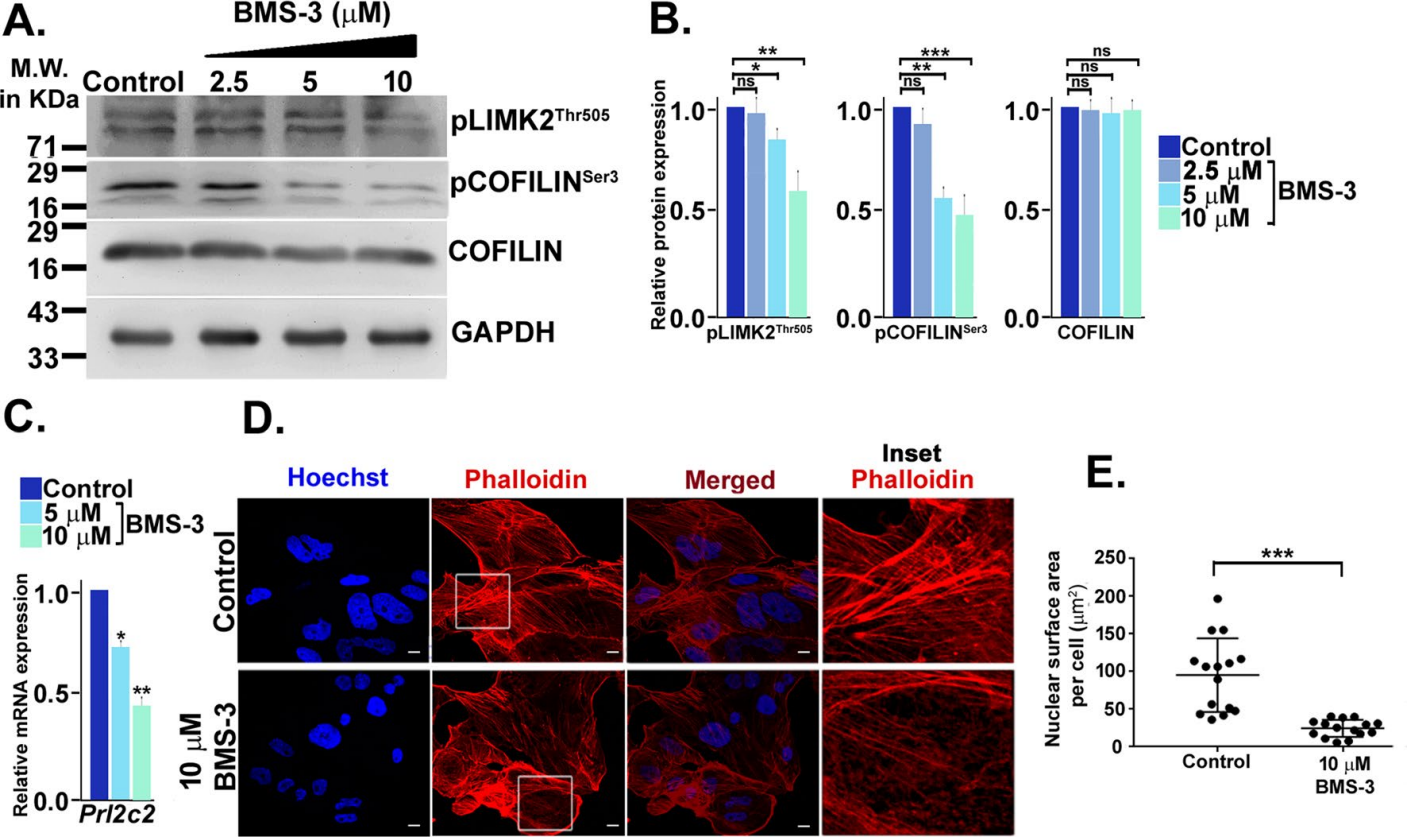
Inhibition of LIMK2 impedes TGC formation. **A** Western blot analysis of pLIMK2^Thr505^, COFILIN, pCOFILIN^Ser3^ in trophoblast cells treated with the indicated doses of BMS-3. Control cells received treatment with equivalent amount of vehicle (DMSO). Cells were harvested after 72 h of treatment. **B** Densitometry-based quantification of the protein bands from **A** using ImageJ. Band intensities of COFILIN were normalized using GAPDH as endogenous control. The level of pLIMK2^Thr505^ and pCOFILIN^Ser3^ was normalized relative to the basal level of the respective proteins. **C** Quantitative real-time PCR analysis of *Prl2c2* transcript in trophoblast cells treated with 5 µM and 10 µM BMS-3. **D** Representative confocal photomicrographs of trophoblast cells under similar experimental conditions. Counterstaining of the nuclei was done using Hoechst (blue). DyLight™ 554 Phalloidin (red) was used to stain the F-actin filaments. Disintegration of the actin filaments has been represented in the boxed areas which have been magnified in the rightmost panel. Scale bar: 10 µm. Magnification: × 72 **D** ImageJ-based quantification of the nuclear surface area/cell of trophoblast cells upon LIMK2 inhibition. Error bars represent the SEM of three independent biological replicates. Statistical analysis was performed using Student’s unpaired *t*-test, *p < 0.05; **p < 0.005; ***p < 0.0005, ns, nonsignificant

To ascertain the importance of LATS1-LIMK2 association in trophoblast development, differentiating trophoblast cells were treated with increasing doses of the LIMK2 inhibitor, BMS-3 (2.5 µM, 5 µM, 10 µM). Although there was no significant change in pLIMK2^Thr505^ and pCOFILIN^Ser3^ levels when cells were treated with 2.5 µM BMS-3, inhibition of LIMK2 activity was confirmed by a significant reduction in pLIMK2^Thr505^ and its substrate pCOFILIN^Ser3^ levels when cells were treated with 5 µM and 10 µM BMS-3 (Fig. 7A, B). Interestingly, treatment of differentiated trophoblast cells with 5 µM and 10 µM BMS-3 significantly decreased TGC marker, *Prl2c2* by 26% and 60%, respectively (Fig. 7C), suggesting that inhibition of LIMK2 using BMS-3 prevented trophoblast differentiation toward the giant cell trajectory. To further validate the cytoskeletal and the nuclear changes upon LIMK2 inhibition, trophoblast cells were subjected to Hoechst and Phalloidin staining post-treatment with 10 µM BMS-3. Impediment of LIMK2 activity mirrored the effects of LATS1 overexpression, with BMS-3-treated cells displaying smaller-sized nuclei (Fig. 7D, E) as compared to control cells. Also, the arrangements of the F-actin filaments were disorganized in BMS-3-treated cells (Fig. 7D) suggesting that inhibition of LIMK2 activity produced phenotypic changes similar to LATS1 overexpression. Thus, our data confirm that LATS1 regulates TGC formation by modulating the LIMK2/COFILIN axis.

**Fig. 8:**
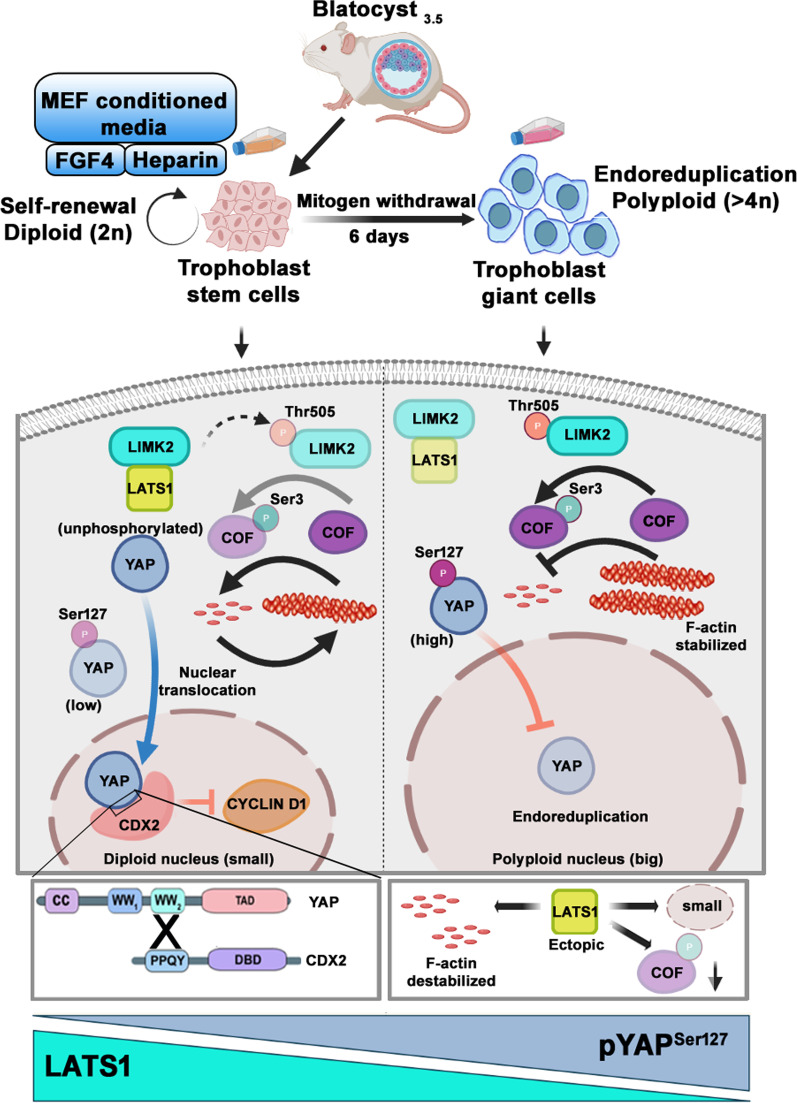
Proposed model of regulation of trophoblast stem cell proliferation and endoreduplication by Hippo. In TSCs, a substantial pool of YAP remains unphosphorylated and translocate to the nucleus. Nuclear YAP sequesters CDX2, thereby controlling trophoblast proliferation by reducing CYCLIN D1 levels. LATS1 in TSCs form LATS1-LIMK2 complex resulting in reduced activation of LIMK2 followed by reduced COFILIN inactivation. On the other hand, phosphorylation of YAP at ser127 prevents it from translocating into the nucleus when TSCs are induced to differentiate. Here, scarcity of LATS1 activates LIMK2 by enhancing pLIMK2Thr^505^ which phosphorylate COFILIN and inhibit it from depolymerizing actin. As a result, endoreduplicated trophoblast giant cell formation occurs

The overall summary of the present study is shown in Fig. 8. In the self-renewing population of murine TSCs, YAP is predominantly unphosphorylated as a result of which it can translocate into the nucleus. The presence of WW_2_ domain in YAP allows it to physically associate with the PPQY domain present in the stemness factor CDX2. Our findings thus suggest the sequestration of CDX2 by YAP as a possible mechanism to control excessive TSC proliferation. On the contrary, phosphorylation of YAP at serine 127 retains YAP in the cytoplasm of differentiated trophoblast cells. Also, the core Hippo kinase LATS1 interacts with LIMK2 in trophoblast cells which is more prominent in TSCs as compared to differentiated trophoblast cells. Further, trophoblast differentiation to TGC is associated with increased LIMK2 activity and consequent COFILIN phosphorylation at serine 3 (inactivation). Shallow LATS1 levels in differentiated trophoblast cells maintain trophoblast endoreduplication and polyploidization by generating a pool of free LIMK2 which is capable of phosphorylating and inactivating COFILIN, thereby preventing it from depolymerizing F-actin. This potentiates the formation of endoreduplicated TGCs.

## Discussion

The regulatory mechanisms manifesting TSC decisions to sufficiently self-renew and/or differentiate dictates the reproductive success during placentogenesis. The molecular episodes governing the expansion and differentiation of TSCs is interdependent on the regulatory cross talks orchestrating signaling cascades to specific transcriptional programs. Numerous recent studies reported the role of Hippo in trophectoderm specification during preimplantation development [27, 28, 52]. Our research initiatives aimed to gain a deeper understanding of Hippo components YAP and LATS1 during TSC self-renewal and differentiation.

This study established a hitherto unknown role of YAP in regulating trophoblast self-renewal. We report that YAP which was previously reported as a shuttling protein [53] serves as an influencer of trophoblast cell fate by dynamically trafficking between the nucleus and cytoplasm during differentiation. Combining immunofluorescence-based localization with cellular fractionation studies, our data revealed the presence of YAP in the nucleus indicating that Hippo is inactive in TSCs. Our data on cytoplasmic retention of YAP in differentiated trophoblast cells suggest that Hippo is active in TGCs. Our observations were further supported by an increase in pYAP^ser127^ levels (signal for cytoplasmic sequestration and inhibition of its activity) upon differentiation [44]. We presume that this phosphorylation creates a binding consensus site for 14-3-3 proteins [44] leading to YAP cytoplasmic retention in differentiated trophoblast cells. Our data are thus in line with the previous finding suggesting the localization of YAP in the nucleus of proliferative cells [54] and cytoplasm of differentiated cells [55, 56]. However, owing to a steady decrease in the phosphorylation status of the primary Hippo kinase responsible for phosphorylating YAP at serine 127, that is, pLATS1^Thr1079^, upon trophoblast differentiation, we believe a complex interplay of other kinases [57, 58] which regulates this dynamicity in YAP phosphorylation during trophoblast differentiation. Thus, unveiling the kinases responsible for phosphorylating YAP at ser127 in differentiated trophoblast cells or the phosphatases which maintains the pool of unphosphorylated YAP in TSCs require further investigation. However, additional molecular mechanisms of YAP regulation by specific cytoplasmic mediators might exist in differentiated trophoblast cells [38, 59]. Therefore, uncovering these non-canonical pathways surrounding YAP which might be functionally segregated from the canonical Hippo signaling pathway needs further study. Our data thus confirms that cytoplasmic retention of YAP in differentiated trophoblast cells serve as an auxiliary mechanism for inhibition of its activity [60]. Therefore, the nucleo/cytoplasmic shuttling of YAP serves as a layer of regulation in such a way that the relative ratio of YAP in the nucleus as compared to the cytoplasm can influence its activity in developmentally indispensable cell types [55, 56].

Our data highlighting a predominant nuclear localization of YAP directly corelate with its functional activity in TSCs. Studies have revealed a significant contribution of YAP in controlling cell proliferation by regulating cell cycle-associated genes [61]. Our BrdU incorporation assay data revealed that under highly confluent cellular environment of cell-to-cell contact inhibition, a scenario which generally observed under in vivo conditions, YAP provides an intrinsic control of trophoblast proliferation even under self-renewing conditions. In addition, our data on the reciprocal regulation of the core TSC G_1_ cyclin, CYCLIN D1 levels, by YAP suggest that depletion of YAP might cause the cells to accumulate in the G_1_ phase of the cell cycle [62]. Thus, we provide an additional mechanism by which YAP may regulate cell cycle. In this regard, YAP may sustain a negative feedback loop to exert a control on excessive proliferation in rapidly proliferating TSCs by restricting cell cycle progression as a context-dependent cellular outcome. Together, these findings signify that YAP might be a potential novel interventional target for hyperplastic trophoblast disorders which remains to be explored.

The obvious dearth of a DNA-binding domain in YAP requires its binding to specific transcription factors to regulate target gene expression [25]. This is primarily mediated by specific binding of YAP to TEAD family of transcription factors [63, 64]. Although the primary function of YAP relies on its localization, an additional mode of regulating YAP to function as a transcription coactivator or corepressor depends on the partner transcription factor. We thus believe that the cellular context and the abundance of the binding partner might play a pivotal role in the choice of its interactors and consequently on the final functional outcome. In this regard, interaction involving the WW domains of YAP with other DNA-binding transcription factors like p73 [65], ERBB4 [66], EGR-1 [67], RUNXs [68] have also been reported which results in context-dependent oncogenic or tumor-suppressive outcomes. Previous research initiatives have highlighted that the switch in YAP-binding partner from growth promoting YAP-TEAD4 to growth suppressing YAP-RUNX3 is associated with reduced tumorigenicity in MKN28 gastric cancer cells [69]. In this report, we highlight a potential binding of YAP to the transcription factor CDX2 in TSCs. It has been previously reported that YAP binds to TEAD during the first cell fate specification in preimplantation embryos promoting CDX2 in the outer polarized cells [70]. However, it remains elusive whether YAP-TEAD influences CDX2 directly. Our data propose an additional regulation of the lineage determining transcription factor of TSC, CDX2. Post-TE specification, that is, under conditions of CDX2 abundance in TSCs, YAP binds to CDX2 itself providing an additional mechanism to regulate uncontrolled proliferation. We hypothesize YAP-dependent CDX2 sequestration as a plausible mechanism to regulate cell proliferation by regulating CYCLIN D1 levels [43]. In addition, to specifically highlight the region utilized in this interaction, our domain deletion experiment confirmed decreased ability of truncated CDX2 lacking the PPQY motif to interact with functional YAP. Thus, our data are in favor of the ability of WW domains in YAP to associate in PPxY motifs present in other DNA-binding transcription factors [8, 65]. Altogether, our data provide a mechanistic example where the core mitotic engine of TSCs is precisely synchronized by the Hippo component YAP.

Our study also contributes to the understanding of the dynamic switch which Hippo employs to critically balance TSC proliferation–differentiation events. As described previously, this is achieved at the first step by cytoplasmic inactivation of YAP. Secondly, our data bring out that the switch from TSC proliferation to endoreduplication is associated with a decrease in the core Hippo kinase LATS1 levels. Thus, the activity of LATS1 is precisely regulated at both translational and post-translational (phosphorylation) levels. Our data showing a decline in pMST^Thr183^ which serves as a signal for autophosphorylation and LATS1 activation [71, 72] suggest that the decline in pLATS1^Thr1079^ is probably due to reduced activation of MST1 during giant cell formation. This further brings out that YAP phosphorylation at ser127 is functionally segregated from the core kinases of the Hippo pathway. However, further studies are necessary to delineate the cross talk of Hippo with other signaling cascades which might operate to balance the replenishment of the stem cell hub along with targeted differentiation toward the giant cell fate. Thus, our results support the transactivation of LATS1 by CUX1, a transcription factor associated with acceleration of S-phase during tumorigenesis [73].

Previous reports suggest that LATS1 houses two distinct domains between amino acid residues 135–353 and 655–755 which can interact with LIM domain containing proteins to modulate actin cytoskeleton [49]. Results from this study shows that LATS1 is capable of forming a complex with LIMK2 in trophoblast cells. In line with the previous finding [49], our data on the activation of LIMK2 (Thr505 phosphorylation on the activation loop) along with the phosphorylation-mediated inactivation of the primary actin-severing protein, COFILIN during trophoblast endoreduplication suggest that LATS1 is a negative regulator of LIMK2 during trophoblast endoreduplication. Low levels of LATS1 maintain the cytokinetic defects in such polyploid population. This inactivation of COFILIN in giant cells by phosphorylation at serine3 thus serves as a mode of regulation by which the giant cells adopt to stabilize actin filaments. Our results therefore highlight that the inactive form of COFILIN results in actin stabilization by reducing actin turnover in giant cells [74]. Decline in the TGC marker gene upon ectopic overexpression of LATS1 during trophoblast differentiation suggests that LATS1 impedes with trophoblast giant cell formation.

In rodents, differentiation of trophoblast giant cells results in a mitotic exit followed by the onset of endoreduplication resulting in a polyploid population with increased DNA content [75]. Reduction in the flow cytometric peaks displaying > 4n DNA content (all of which must be giant cells) in LATS1 overexpressing differentiated trophoblast cells confirmed LATS1 as a novel negative modulator of trophoblast giant cell fate. This was also evident from our confocal photomicrographic images displaying smaller-sized nuclei in LATS1 overexpressing cells. The disintegration of F-actin in LATS1 overexpressing trophoblast cells suggests that ectopic overexpression of LATS1 collapses the F-actin cytoskeletal network and lower levels of LATS1 serves to stabilize actin filaments in trophoblast giant cells. Thus, our study supports the previous findings suggesting LATS1 as a negative regulator of actin polymerization and loss of LATS1 leads to cytokinesis failure [49, 76]. At the molecular level, our data highlight that LATS1 negatively regulates actin polymerization by decreasing LIMK2 activity. Proteins that function via phosphorylation are generally reverted back to their unphosphorylated state by phosphatases. However, unaltered levels of the LIMK2 phosphatase, CHRONOPHIN further confirms that reduced COFILIN inactivation in LATS1 overexpressing cells is due to reduced activation of LIMK2 and not via increased activation of CHRONOPHIN. Our data on LATS 1 overexpression were reaffirmed by BMS-3-dependent inhibition of LIMK2. Thus, our study highlights that endoreduplication of trophoblast cells is associated with a coordination of LIMK2 with the actin-severing protein COFILIN. Altogether, we establish a LATS1-LIMK2-COFILIN axis where ectopic LATS1 inhibits the kinase activity of LIMK2 to decrease the pool of inactive COFILIN and its effect on actin stabilization. It appears that the disintegration of actin filaments in LATS1 overexpressing trophoblast cells is due to LATS1-dependent reduction in pLIMK2^Thr505^ and pCofilin^Ser3^ levels. Our study, is thus, in line with the previous findings suggesting major actin-dependent cytoskeletal changes during giant cell formation [77].

## Conclusion

These data unveil a dual regulatory mechanism by which Hippo tips the balance between TSC self-renewal and endoreduplication. Dynamic nucleo-cytoplasmic shuttling of the terminal Hippo component YAP upon TSC differentiation is evident. Furthermore, differentiation-dependent increase in pYAP^Ser127^, the hallmark of cytoplasmic sequestration is established. Domain deletion experiments revealed that nuclear YAP present in TSCs utilizes its WW_2_ domain to directly interact with the PPQY motif of CDX2. Loss of and gain in function of YAP suggested YAP-mediated sequestration of CDX2 and decreased CYCLIN D1 levels as a plausible mechanism to control TSC proliferation. Shallow levels of the core Hippo kinase LATS1 is imperative to trophoblast giant cell formation. A potential regulatory role of LATS1-LIMK2 complex in trophoblast cells has been demonstrated. Low levels of LATS1, increased LIMK2 and pLIMK2^Thr505^ leads to phosphorylation of COFILIN at Ser3, thereby impeding its activity in trophoblast giant cells. Precocious overexpression of LATS1 resulting in a substantial decrease in the giant cell marker *Prl2c2,* inhibition of endoreduplication by reducing ploidy, disintegrated actin filaments as well as nuclear size reaffirms its function in trophoblast cells.

## Supplementary Information

**Additional file 1.** Supplementary figures.

**Additional file 2.** Supplementary materials and method.

## Data Availability

Data and materials are available on request from the corresponding author.

## Abbreviations

YAP: Yes-associated protein
LATS1: Large tumor suppressor kinase 1
TSC: Trophoblast stem cell
CDX2: Caudal type homeobox 2
LIMK2: LIM domain kinase 2
TGC: Trophoblast giant cells
TE: Trophectoderm
ICM: Inner cell mass
FGF4: Fibroblast growth factor 4
FBS: Fetal bovine serum
MEF: Mouse embryonic fibroblast
ATCC: American type culture collection
qPCR: Quantitative real-time PCR
RT-PCR: Reverse transcriptase PCR
BrdU: 5-Bromodeoxyuridine
