# Supplementary material for: Molecular regulation of trophoblast stem cell self-renewal and giant cell differentiation by the Hippo components YAP and LATS1
Source: Stem Cell Res Ther. 2022 May 7;13:189. doi: 10.1186/s13287-022-02844-w (PMC9080189; doi:10.1186/s13287-022-02844-w)
Supplement: Supplementary file 1 — Additional file 1. Supplementary figures. [file 13287_2022_2844_MOESM1_ESM.doc]

**MATERIALS AND METHOD**

***Cell culture***

Murine blastocyst derived TSCs (TS_3.5_) were a kind gift from Professor Janet Rossant, The Hospital for Sick Children (SickKids), Toronto, Canada and cultured as previously reported [11,38,39,40]. TSCs were sustained in stemness media containing 3:7 ratio of TS basal media [RPMI-1640 (Sigma Aldrich) supplemented with 20% FBS, 1% Penicillin-streptomycin, 1% Glutamax (Thermo Fisher), 1mM sodium pyruvate and 100µM β-mercaptoethanol (Sigma Aldrich)] : mitotically inactivated mouse embryonic fibroblast (MEF) conditioned media, supplemented with 25ng/ml FGF4 and 1μg/ml heparin. Differentiation was induced by the withdrawal of FGF4, heparin and MEF conditioned media followed by growth in TS basal media and cultured for 6 consecutive days which allows differentiation to the default giant cell trajectory. TSCs and differentiated trophoblast cells were characterized as described previously [38,41]. Subculturing was done by trypsinisation with 0.05% Trypsin-EDTA (Gibco) for 20 seconds. Cells were maintained under a continuous supply of 5% CO_2_ at 37°C in a humidified incubator.

SH-SY5Y cells were maintained in a 1:1 mixture of Eagle’s Minimal Essential Medium (Sigma Aldrich) and F12K Ham supplemented with 10% FBS (Invitrogen), 1% Penicillin-streptomycin (Gibco), 1% Glutamax (Gibco) and 1 mM sodium pyruvate (Sigma Aldrich). Sub-culturing was done using 0.25% Trypsin-EDTA (Gibco) at 70% confluence.

***Cloning and characterization of full-length mouse YAP, CDX2, LATS1 and deletion constructs of YAP and CDX2***

Template cDNA for cloning of mouse YAP (NM_001171147.1), CDX2 (NM_007673.3) and LATS1 (NM_010690.1) was obtained by reverse transcription of mouse TSC RNA using M-MLV Reverse transcription kit (Invitrogen). LA Taq DNA Polymerase (TaKaRa) was used for amplification of full-length constructs and deletion mutants. Three independent deletion mutants of YAP (ΔWW_1_, ΔWW_2_ and ΔWW_1_WW_2_) and one deletion mutant of CDX2 (ΔCDX2) were constructed. ΔWW_1_, ΔWW_2_ and ΔCDX2 were cloned by ligating two different amplified fragments whereas ΔWW_1_WW_2_ was cloned by ligating three different fragments. The amplified cDNA was cloned in p3XFLAG-CMV™-10 expression Vector (Sigma Aldrich) and one Shot Mach1 T1 cells (Invitrogen) were used for transformation. All the primers and restriction enzymes used for cloning have been enlisted in Table 1. Clone confirmation of was done using restriction digestion and Sanger’s di-deoxy sequencing of the inserts.

***Transient transfection of siRNAs and plasmid***

In order to down regulate endogenous YAP in TSCs, two pre-validated silencer-select siRNAs [(s202423 and s76160), Ambion] targeting the coding region of YAP was transfected using Lipofectamine RNAiMax (Invitrogen) at 60% cell confluence. Cells were initially treated with three independent doses of siYAP cocktail (20nM, 100 nM, 200 nM). A dose of 100nM siYAP (50nM each siRNA) was selected for further experimentation based on the maximum down-regulation obtained by quantitative real-time PCR. Cells treated with equal amount of scrambled siRNA were used as control.

Depending on the experimental requirement of functional and interaction studies, 2.5µg of either full-length or deletion constructs of YAP and LATS1 cDNA constructs was ectopically overexpressed in TSCs using Lipofectamine 2000 (Invitrogen) at 70% cell confluence. For YAP over-expression, stemness was maintained after 6 hours of transfection and cells were harvested after 48 hours whereas, for LATS1 overexpression, differentiation was induced 6 hours post transfection and cells were harvested after 72 hours.

SH-SY5Y cells were transfected either with full-length YAP or CDX2 cDNA construct or the cDNA construct expressing the deleted variants of YAP and CDX2 using Lipofectamine 2000 (Invitrogen) at 70% cell confluence. For interaction studies involving CDX2 deletion constructs, 1.5µg of full-length CDX2 and ΔCDX2 constructs were independently co-transfected with 1.5µg of full-length YAP cDNA construct. Stemness was maintained after 6 hours of transfection. Control cells were transfected with empty vector backbone at a similar concentration and the cells were harvested after 48 hours of transfection.

***RNA extraction, reverse transcription, PCR and quantitative real-time PCR***

RNA isolation was carried out using cold TRIzol reagent (Ambion) as per the manufacturer’s protocol. For isolation of RNA from trophoblast stem cells, cells were washed with DPBS and trypsined with 0.05% Trypsin-EDTA (Gibco) for 20 seconds to eliminate the contaminating differentiated cells. The cell pellet obtained after trypsinization was washed with PBS (Gibco) and used for RNA isolation. For isolation of RNA from differentiated trophoblast cells, cell monolayers were directly washed with PBS (Gibco) and scraped off after addition of TRIzol directly to the culture dishes. A total of 5μg RNA was used to reverse transcribe first-strand cDNA using oligo dT primers (Invitrogen) and M-MLV reverse transcriptase kit (Invitrogen).

Prior to sequencing the clones for correctness, deletion mutants of YAP and CDX2 was verified by RT-PCR using PCR master mix (Thermo Scientific) and 1μl of the first-strand cDNA with 400nM each of YAP forward (5’- GCAGTCCTCCTTTGAGATCCCT-3’) and reverse (5’-GCAAAACGAGGGTCCAG CCTT-3’) and CDX2 forward (5’- CTGAAACCTGGCTCCGCAGAA -3’) and reverse (5’-TGATGCGGGTGATGGTGCG-3’). The PCR conditions included an initial denat-uration at 95ºC for 2 minutes followed by a denaturation at 95ºC for 30 seconds, annealing at 57ºC for 30 seconds and extension at 72ºC for 1 minute followed by final extension of 72ºC for 7 minutes.

FG-Power SYBR Green master mix (Applied Biosystems) and a ten-fold dilution of individual cDNA were used for real time PCR analysis of different genes using the 7500 Real-time PCR system (Applied Biosystems). The conditions included an initial holding stage (95°C for 10 minutes) and 40 amplification cycles (95°C for 15 seconds and 60°C for 1 minute) followed by a dissociation stage (95°C for 15 seconds, 60°C for 1 minute and 95°C for 30 seconds). The primers used are listed in Table 2 and were designed using a software Primer express 3.0.1 supplied with the Real Time PCR machine. *rPL7* was used as an endogenous control for normalized gene expression.

***Western Blot Analysis***

Western Blotting was performed as described previously [38-42]. TSCs were mildly trypsined with 0.05% Trypsin-EDTA (Gibco) for 20 seconds and differentiated cells were scraped for total protein isolation. Cells were lysed in RIPA buffer containing 20mM Tris-HCl (pH 7.5), 150mM sodium chloride supplemented with 1% NP-40, 1mM disodium EDTA, 1mM freshly prepared EGTA, 0.2mM freshly prepared PMSF, 1X protease inhibitor cocktail (Cell Signalling Technologies) and 1X phosphatase inhibitor cocktail (Cell Signalling Technologies). The concentration of individual samples was estimated using Bio-Rad protein assay reagent (Bio-Rad, Hercules). Protein samples were resolved on a 10% SDS-PAGE and transferred onto PVDF membrane (Millipore). Signal detection was done using an enhanced chemiluminescence reagent, Luminata Forte (Millipore). Images were captured using the ChemiDoc imaging system (UVP) and the band intensities were quantified using ImageJ software (NIH, USA).

***Immunoprecipitation***

For immunoprecipitation, 250μg cell lysate was incubated overnight at 4ºC with the desired capture antibody under continuous mixing to allow the formation of antigen-antibody complex. The preformed antigen-antibody complex was captured using Pure Proteome Protein A/G Magnetic Beads (Millipore) with continuous mixing for 2 hours at room temperature and immunoprecipitation was performed as per manufacturer’s protocol.

***Immunofluorescence***

Immunofluorescence was performed on cells plated onto coverslips. For TSCs and differentiated cells, TSCs were directly plated on coverslips and stemness was maintained or differentiation was induced depending on the experimental requirement. However, for transfected cells, trypsinization was done with 0.05% Trypsin-EDTA (Gibco) 24 hours post transfection and equal number of cells were plated on coverslips. Cells were fixed using ice-cold 4% paraformaldehyde and blocking was done in blocking buffer (PBS pH 7.4 containing 5% normal goat serum and 0.3% Triton-X) followed by incubation with the respective antibodies in antibody dilution buffer (PBS containing 1% BSA and 0.3% TritonX) at room temperature. Post incubation with primary antibody, cells was washed thrice with PBS followed by incubation with TRITC conjugated anti-mouse IgG (Sigma Aldrich) under complete darkness. Nuclear-counterstaining was performed using Hoechst (2µg/ml) and images were acquired using FV10i Confocal Laser-scanning microscope (Olympus). ImageJ software was used for the quantification of CTCF [(Corrected Total Cell Fluorescence), CTCF = Integrated Density of each cell − (Area of selected cell × Mean fluorescence of background readings). Imaging was done with FV10i Confocal Laser-scanning micro-scope (Olympus).

***Hoechst and Phalloidin staining of F-actin***

For Hoechst and Phalloidin staining under LATS1 overexpressing conditions, TSCs were seeded onto 35mm dishes. At 70% confluence, cells were transfected with cDNA constructs to ectopically overexpress LATS1. Control cells were transfected with empty vector backbone. For Hoechst and phalloidin staining under LIMK2 inhibition, cells were seeded under differentiating conditions. Cells were then treated with 10 µM BMS-3 for 6 hours. Control cells were treated with vehicle (DMSO). For both the experiments, cells were induced to differentiate by culturing in TS complete media after 6 hours. 24 hours thereafter, cells were treated by harsh trypsinization using 0.25% Trypsin-EDTA (Gibco) and equal number of cells were plated onto coverslips in a 35mm dish. Cells were harvested 48 hours thereafter.

Cells were harvested by fixing with 4% paraformaldehyde, washed thrice with sterile DPBS and stained with Phalloidin (Cell Signaling Technology, #13054) diluted in methanol at concentration of 1:200. Post washing, nucleus was counterstained with Hoechst at a final concentration of 2μg/ml for 20 minutes. Imaging was done with TCS SP8 Confocal microscope (Leica). Nuclear surface area was quantitated using Image J software.

***5-Bromodeoxyuridine incorporation assay***

TSCs were transiently transfected with a) either siYAP or scramble siRNA for YAP down regulation experiments, b) either YAP-cDNA or control empty vector for ectopic overexpression. Cells were maintained in stemness media 6 hours following transfection. The cells were trypsined after 24 hours of transfection and seeded at a density of 50,000 cells/well in 200µl with BrdU containing medium (1:1000) in a 96 well plate. Cells were harvested after 24 hours and BrdU incorporation was assessed as per manufacturer’s instruction of the BrdU cell proliferation assay kit (Cell Signaling Technologies) as described previously [43].

To assess BrdU incorporation by immunofluorescence, equal number of cells were seeded onto coverslips in 35mm dishes. BrdU incorporation assay was performed described before and then cells were fixed. BrdU incorporation was detected using TRITC conjugated goat anti-mouse IgG and the fluorescence intensity was quantitated as described previously [43].

***Polyploidy analysis by flow cytometry***

TSCs were transfected with either empty vector (control) or LATS1-cDNA. Differentiation was induced 6 hours post transfection and cells were thereafter cultured for 72 hours. Cells were harvested by harsh trypsinization using 0.25% Trypsin-EDTA (Gibco) to allow detachment of the giant cells. The cell pellet was resuspended and washed twice with PBS. The cell pellet was washed and resuspended in DPBS and a suspension of 10^6^ cells/ml was prepared in 250µl cell volume and stained with Hoechst solution at a final concentration of 2µg/ml by incubating at 37ºC for 30 minutes. TSCs were used for cell gating and the ploidy was analyzed by measuring cell fluorescence using a flow cytometer (LSR Fortessea, BD).
