# Supplementary material for: Molecular regulation of trophoblast stem cell self-renewal and giant cell differentiation by the Hippo components YAP and LATS1
Source: Stem Cell Res Ther. 2022 May 7;13:189. doi: 10.1186/s13287-022-02844-w (PMC9080189; doi:10.1186/s13287-022-02844-w)

**Molecular regulation of trophoblast stem cell self-renewal and giant cell differentiation by the Hippo components YAP and LATS1**

**Trishita Basak^1^ and Rupasri Ain^1,*^**

^1^Division of Cell Biology and Physiology, CSIR-Indian Institute of Chemical Biology, Kolkata, India.


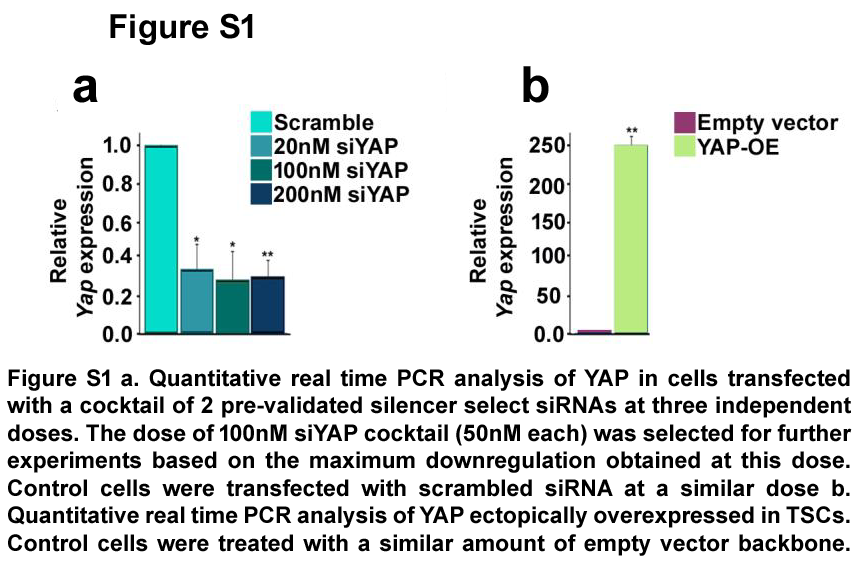


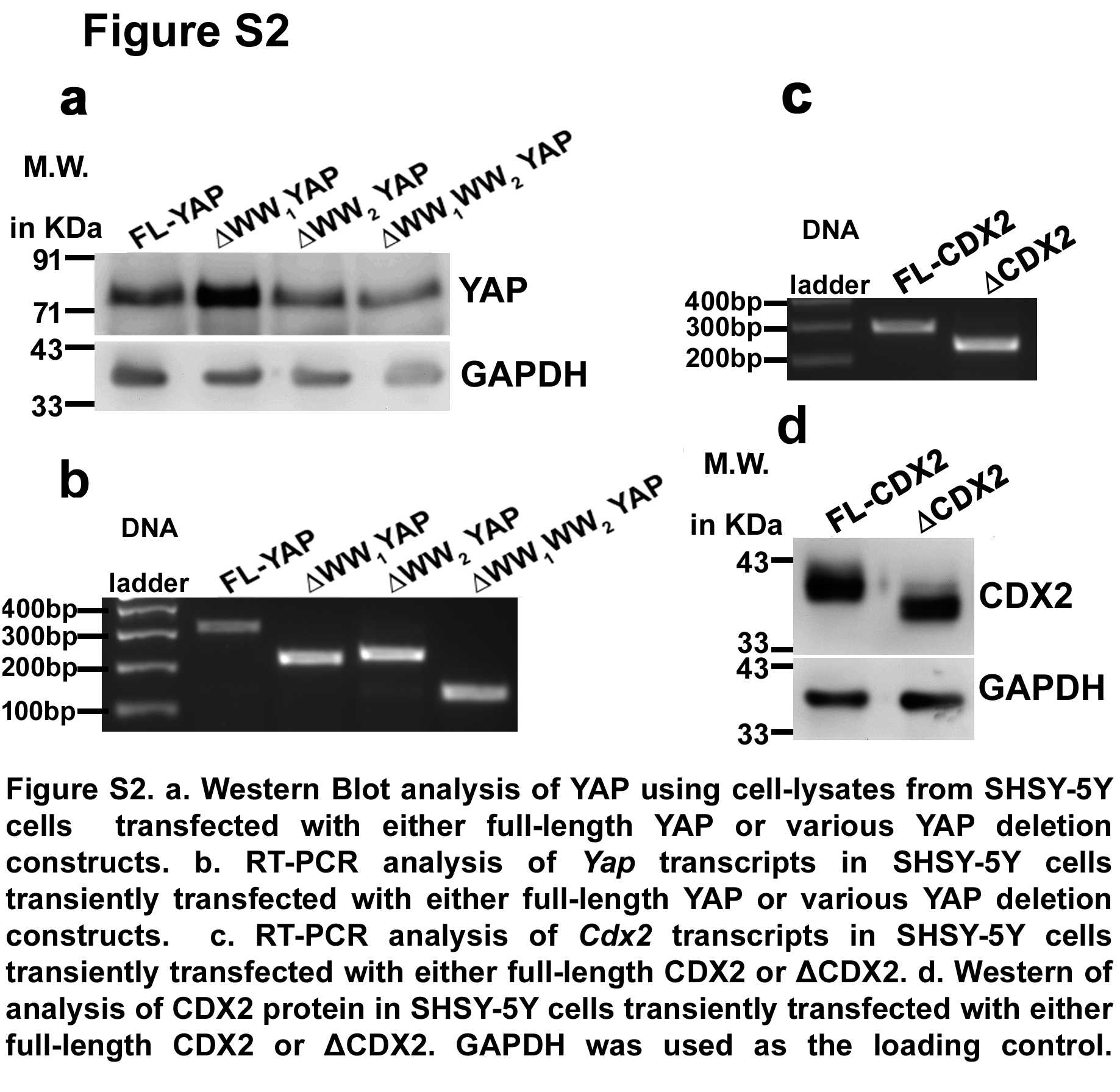


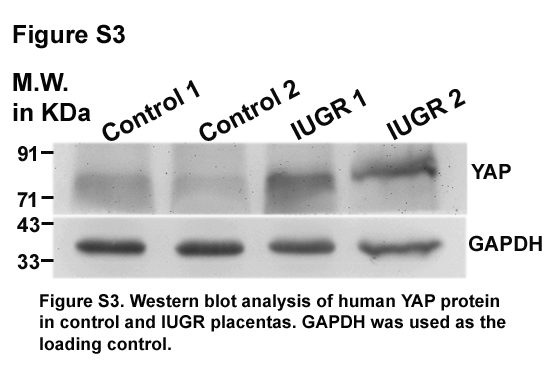


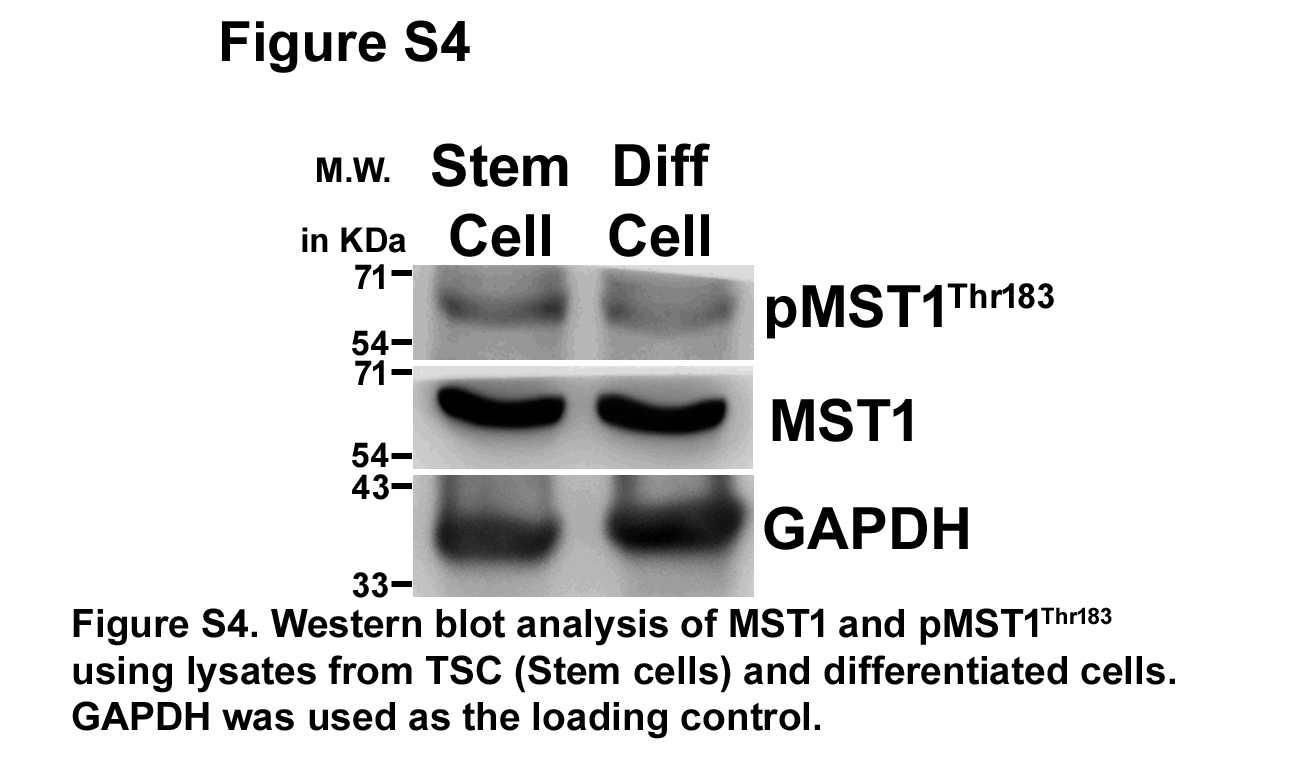

Supplement: Supplementary file 2 — Additional file 2. Supplementary materials and method. [file 13287_2022_2844_MOESM2_ESM.doc]
